# From water to sediment: A meta-analysis of microplastic distribution and the impact of dams in reservoir ecosystems

**DOI:** 10.1016/j.eehl.2025.100188

**Published:** 2025-09-26

**Authors:** Wei Gao, Peng Zhang, Hongcui Wang, Xiaohan Yang, Chunjiang An

**Affiliations:** a Department of Building, Civil and Environmental Engineering, Concordia University, Montreal, Quebec H3G 1M8, Canada; b Center of Eco-environmental Monitoring and Scientific Research, Administration of Ecology and Environment of Haihe River Basin and Beihai Sea Area, Ministry of Ecology and Environment of the People's Republic of China, Tianjin 300211, China; c Institute for Energy, Environment and Sustainable Communities, University of Regina, Regina, Saskatchewan S4S 0A2, Canada

**Keywords:** Microplastics, Reservoirs, Dam trapping effect, Meta-analysis, Statistical modeling, Ecological impact

## Abstract

Microplastics (MPs) have become major contaminants in freshwater ecosystems. While numerous studies have characterized MPs in reservoirs, a comprehensive synthesis focusing on in-reservoir variations and dam-related influences is still lacking. This study investigates the spatial distribution of MPs in reservoir water and sediment based on data synthesized from 34 peer-reviewed studies covering 36 reservoirs across diverse climatic and hydrological regions worldwide, with a focus on the trapping effects of dams. Using a combination of generalized linear mixed models (GLMM) and generalized additive mixed models (GAMM), the study analyzed MP abundance patterns in relation to distance to the dam and identified key environmental and anthropogenic factors influencing their distribution. The results show that MPs tend to accumulate near dams, suggesting a trapping effect, while upstream MP concentrations decline with increasing distance. Vertical stratification patterns were observed in both water and sediment, indicating different transport mechanisms. Additionally, exposure to MPs significantly affected benthic organisms, particularly in terms of growth and reproduction, with effects intensifying over longer exposure durations. These findings highlight the need for improved monitoring and management strategies in reservoirs to mitigate MP pollution and its ecological consequences.

## Highlights

• Microplastics accumulate near dams, indicating a trapping effect in reservoirs.
• Microplastic concentrations decline with increasing distance from the dam in upstream regions.
• Vertical distribution of microplastics is influenced by a range of environmental factors.
• Microplastic exposure impairs the growth and reproduction of benthic organisms over time.
• Dams alter microplastic transport in reservoirs, posing long-term ecological risks.

## Graphical abstract

**Figure:**
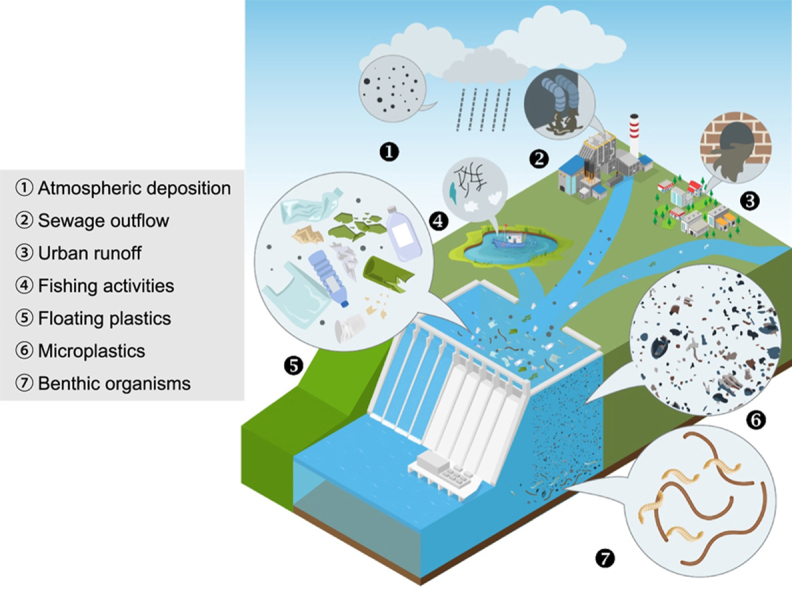

## 1 Introduction

Dams and reservoirs alter natural hydrodynamics, significantly influencing the distribution of suspended particles and associated pollutants in both horizontal (upstream-downstream) and vertical (depth-related) dimensions [1]. Horizontally, these man-made structures often trap suspended particles, leading to the accumulation of pollutants, such as nutrients and heavy metals, in specific reservoir zones, while downstream areas may experience reduced pollutant loads. For example, the trapping of nutrients can cause eutrophication within the reservoir, whereas decreased nutrient flows downstream (particularly in deltas and floodplains) may result in oligotrophication [2,3]. Similarly, metal concentrations in sediment near dams in a canyon reservoir were significantly higher than those upstream, indicating that potential ecological risks are strongly correlated with proximity to the dam [4]. Vertically, reservoirs often exhibit thermal stratification, forming distinct biochemical layers with depth [5,6]. In large hydropower systems such as Hoover Dam, water is frequently discharged from deeper, anoxic layers to meet power generation needs, potentially altering downstream thermal regimes and inducing hypoxic stress [2,6]. These patterns of physical stratification and discharge not only influence conventional pollutants but may also affect the transport and fate of emerging contaminants such as microplastics (MPs).

Unlike nutrients and metals, MPs present a novel challenge due to their particle-based nature and diverse physicochemical properties, including variations in composition, morphology, size, and color. These properties govern their environmental behavior, enabling them to adsorb hydrophobic pollutants and act as carriers for microbial colonization through biofilm “plastispheres” [7,8]. In freshwater, MPs mainly come from domestic wastewater, industrial effluents, surface runoff, and atmospheric fallout, with fragments and fibers being most common. Abundances vary from tens to tens of thousands particles per m^3^. Although MP pollution has been studied earlier in marine environments, its significance in freshwater systems, including rivers, lakes, lagoons, and reservoirs, is also increasingly recognized [[9], [10], [11]]. These freshwater ecosystems act as both sinks and pathways for MPs, which are redistributed between environmental compartments, from terrestrial sources to freshwater and ultimately marine environments [[12], [13], [14]]. Despite growing recognition of MPs in freshwater systems, the specific role of dam–reservoir systems in modulating MP fate remains poorly quantified.

In particular, reservoirs and dams play a key role in shaping the fate and transport of MPs in freshwater systems by regulating water flow and sediment deposition. Dams can increase water retention time and reduce flow velocity, enhancing the sedimentation of suspended solids, including MPs. Unlike rivers, which rapidly transport MPs downstream, or lakes with more stable but natural stratification, reservoirs exhibit unique flow regulation and retention patterns due to dam operations, making them distinct in both MP accumulation and remobilization dynamics. For example, Zhang et al. [15] reported MP abundances as high as 13,617.5 ​× ​10^3^ items/km^2^ in the surface water of the Three Gorges Reservoir in China, suggesting that damming can create hotspots for MP accumulation. In addition to the trapping effects specific to dam structures, reservoirs share common features with lakes and rivers in MP dynamics. MP distribution in freshwater systems is heavily influenced by terrestrial sources and hydrodynamic conditions [16], often leading to localized accumulation in low-flow areas. In many regions, high population density and inadequate waste management increase the risk of MP pollution in inland waters [17]. Furthermore, lower salinity and density in freshwater systems make MPs more likely to settle [18], and the relatively short and simple food chains in freshwater ecosystems facilitate the entry of MPs into the food web [19].

Although many studies have explored MPs, a systematic assessment of MP behavior and its key drivers in reservoirs is still lacking [20]. On a macro level, considering each reservoir as a whole can help to determine the global fate and distribution of MPs in dam–reservoir systems. At the individual reservoir level, attention shifts to understanding how dams, as hydraulic structures, influence MP dynamics by analyzing the differences in abundance before and after the dam and examining the effects of both horizontal and vertical water movements on MP distribution. Additionally, examining MP behavior across cascade reservoir systems, including their movement through connected rivers, provides valuable insights into their longitudinal transport [21]. Beyond transport and distribution, MPs can also adversely affect freshwater organisms, particularly their survival, growth, and reproduction, which raises concerns about ecosystem health and biodiversity [[22], [23], [24]]. To address these research gaps, this study aims to (1) investigate the global distribution characteristics of MPs in dam–reservoir systems, (2) quantify the relative contributions of key factors influencing MPs based on models and functional analysis, (3) determine the effects of MPs on freshwater organisms, particularly benthic species, and (4) explore effective management strategies to mitigate the presence and impact of MPs in reservoirs.

## 2 Materials and methods

### 2.1 Data sources

A systematic literature search of the peer-reviewed studies on reservoir MPs was conducted in the Web of Science database in June 2024. The search was performed using the following search string: (“microplastic” OR “microplastics”) AND (“dam” OR “reservoir”). A total of 440 articles were retrieved and imported into EndNote X9 to remove duplicates. Furthermore, the articles were manually screened based on the following criteria: (1) The study must be related to the dam–reservoir. Off-stream or natural reservoirs without traditional dams or dams on rivers were excluded from the analysis. (2) The location of both the dam and the sampling sites must be provided in the article to ensure data accuracy. (3) Articles that presented MP concentration per unit area (items/km^2^) were excluded. (4) The study must explore MP concentrations in either water or sediment. (5) At least one characteristic of MPs, such as abundance, color, composition, or shape, must be mentioned in the article. Following this screening process, 34 articles were selected, covering 36 reservoirs (Appendix S1). In some cases, certain cascade dam systems included multiple smaller reservoirs. For simplicity, we counted cascade reservoirs from the same study as a single reservoir. Reported data on MP characterization were extracted directly from the tables or figures using GetData Graph Digitizer software. The population density was collected from GPW version 4 with a resolution of 1 ​km [25]. The surface area was preferentially derived from the respective study, and if not reported in the literature, information collected from the Global Dam Watch database version 1.0 was used [26]. ArcGIS 10.8 (ESRI, Redlands, CA, USA) was used for spatial data processing and mapping.

### 2.2 Data analysis

To investigate the impact of anthropogenic and environmental factors on MP abundance in reservoir water and sediment, the adjusted dataset was analyzed using both generalized additive mixed models (GAMM) and generalized linear mixed models (GLMM). Both models incorporate random effects (specifically, reservoir identifier) to account for inter-reservoir heterogeneity, enhancing the accuracy and generalizability of the results. GLMMs were used to evaluate overall differences in MP abundance between upstream and downstream locations, as well as to assess linear relationships within spatially constrained regions (e.g., within 100 ​km upstream of dams) [27,28]. In contrast, GAMMs were applied to the full upstream dataset to explore broader and potentially nonlinear patterns in MP distribution [29,30].

GAMM is a flexible regression model that combines linear effects, smooth functions of covariates, and random effects, making it well-suited for capturing complex relationships. We implemented the GAMM approach using the mgcv package in R [31] to identify key factors influencing MP abundance. The model could be expressed as:

$$g\left(\mu_{i}\right)=\beta_{0}+\sum_{j}\beta_{j}x_{ji}+\sum_{k}f_{k}x_{ki}+b_{{site}_{i}}+b_{{site}_{i}}^{\left(Dis\right)}{Dis}_{i}+\epsilon_{i}$$

where, $g\left(\mu_{i}\right)$ indicates the model's simulation results (log link function for the negative binomial distribution), $\beta_{0}$ is the overall intercept, $\sum_{j}\beta_{j}x_{ji}$ represents the linear effect of predictors, and $\sum_{k}f_{k}x_{ki}$ represents the smooth functions of covariates to capture potential nonlinear relationships. The term $b_{{site}_{i}}$ is the site-level random intercept, capturing reservoir-specific variability in MP abundance, and $b_{{site}_{i}}^{\left(Dis\right)}{Dis}_{i}$ is the random slope for distance to the dam (Dis), allowing the effect of distance to vary across reservoirs. The term $\epsilon_{i}$ represents the residual error.

The GLMM follows a similar structure to the GAMM but assumes that all independent variables have a linear relationship with MP abundance. It could be expressed as:

$$g\left(\mu_{i}\right)=\beta_{0}+\sum_{j}\beta_{j}x_{ji}+b_{{site}_{i}}+\epsilon_{i}$$

where, the terms have the same interpretations as in GAMM, except that there are no smooth $f_{k}x_{ki}$ functions, meaning that all covariates contribute additively in a linear fashion.

The GLMM was fitted using the glmmTMB package [32]. Initially, we tested models with both random intercepts and random slopes (1 ​+ ​Dis ∣ Site). However, the variance associated with the random slope for Dis was negligible, and model comparisons based on Akaike Information Criterion (AIC) values indicated that a simpler random-intercept model (1 ∣ Site) provided an equally good fit while reducing model complexity.

Given that the integrated data exhibited overdispersion and did not conform to the normality assumptions, we applied a negative binomial model (nbinom2) for both GAMM and GLMM, utilizing a logarithmic link function for modeling count data, and statistical significance was assessed at α ​= ​0.05. Model fit was assessed using simulated residual diagnostics with the DHARMa package [33], ensuring that residual patterns did not indicate violations of model assumptions. Model predictions were computed as estimated marginal means using the ggeffects package [34], and data visualization was performed with the ggplot2 package [35]. All analyses were conducted in R (v4.3.3). Five predictors were selected based on their known or potential relevance to MP transport and accumulation in reservoirs. Distance to the dam was included to represent spatial variation in hydrodynamics and sedimentation. Population density was used as a proxy for anthropogenic pressure, particularly plastic waste input. Surface area served as a morphometric parameter, influencing water retention time and mixing. Water sampling method and mesh filter size were included to account for differences in data collection procedures that could affect MP detection.

In addition to GLMM and GAMM, several supplementary analyses were conducted. A distance decay analysis was applied to assess how similarities in MP composition declined with increasing geographic distance between sampling sites. Furthermore, for sites where both sediment and water samples were collected, non-parametric correlation tests (Spearman's and Kendall's correlation tests) were used to evaluate the relationship between MP abundance in water and sediment.

### 2.3 Theoretical modeling of vertical settling velocity

The vertical transport of MPs is classically described by Stokes' law (Eq. 1) [36], which assumes laminar flow and neglects thermal stratification. To adapt this to reservoir conditions, we proposed two critical modifications. First, under surface turbulence (Re ​> ​2000), enhanced vertical mixing requires modifying the drag coefficient (*C*_*d*_) in Eq. (1), transitioning from Stokes' laminar flow assumption (*C*_*d*_ ​= ​24/*R*_*e*_) to a turbulence-adapted formulation [37]. Second, water density (*ρ*_*w*_) and viscosity (*μ*) vary with depth-dependent temperature (*T*) [38,39]. We integrate empirical temperature functions (Eqs. (2), (3))) into the settling velocity calculation. These lead to a comprehensive model (Eq. 4) accounting for both turbulence and thermal effects.

(1) $$v=\frac{\left(\rho_{\omega }-\rho_{p}\right)gd^{2}}{18\mu }$$

(2) $$\rho_{\omega }\left(T\right)=\frac{999.8395+16.9452T-0.0080T^{2}-4.6170\times 10^{-5}T^{3}}{1+0.0169T}$$

(3) $$\mu \left(T\right)=10^{-3}\times e^{-3.7188+\frac{578.919}{135.604+T}}$$

(4) $$v\cdot \left|v\right|\left(\frac{24\mu \left(T\right)}{\rho_{\omega }\left(T\right)d\left|v\right|}+5\sqrt{\frac{\mu \left(T\right)}{\rho_{\omega }\left(T\right)d\left|v\right|}}+\frac{2}{5}\right)=\frac{4}{3}\frac{\left(\rho_{\omega }\left(T\right)-\rho_{p}\right)gd}{\rho_{\omega }\left(T\right)}$$

where, *μ* (Pa·s) and *ρ*_*w*_ (kg/m^3^): water viscosity and water density as a function of temperature; *d* (m): MP diameter; *g* (m/s^2^): gravitational acceleration; *ρ*_*p*_ (kg/m^3^): particle density; v (m/s): terminal velocity. The parameter *d* ranges from 10^−6^ to 10^−3^ ​m (1 ​μm–1 ​mm), and the parameter *ρ*_*p*_ was set from 800 to 2000 ​kg/m^3^.

## 3 Results and discussion

### 3.1 Global distribution characteristics of MPs in reservoirs

**Fig. 1:**
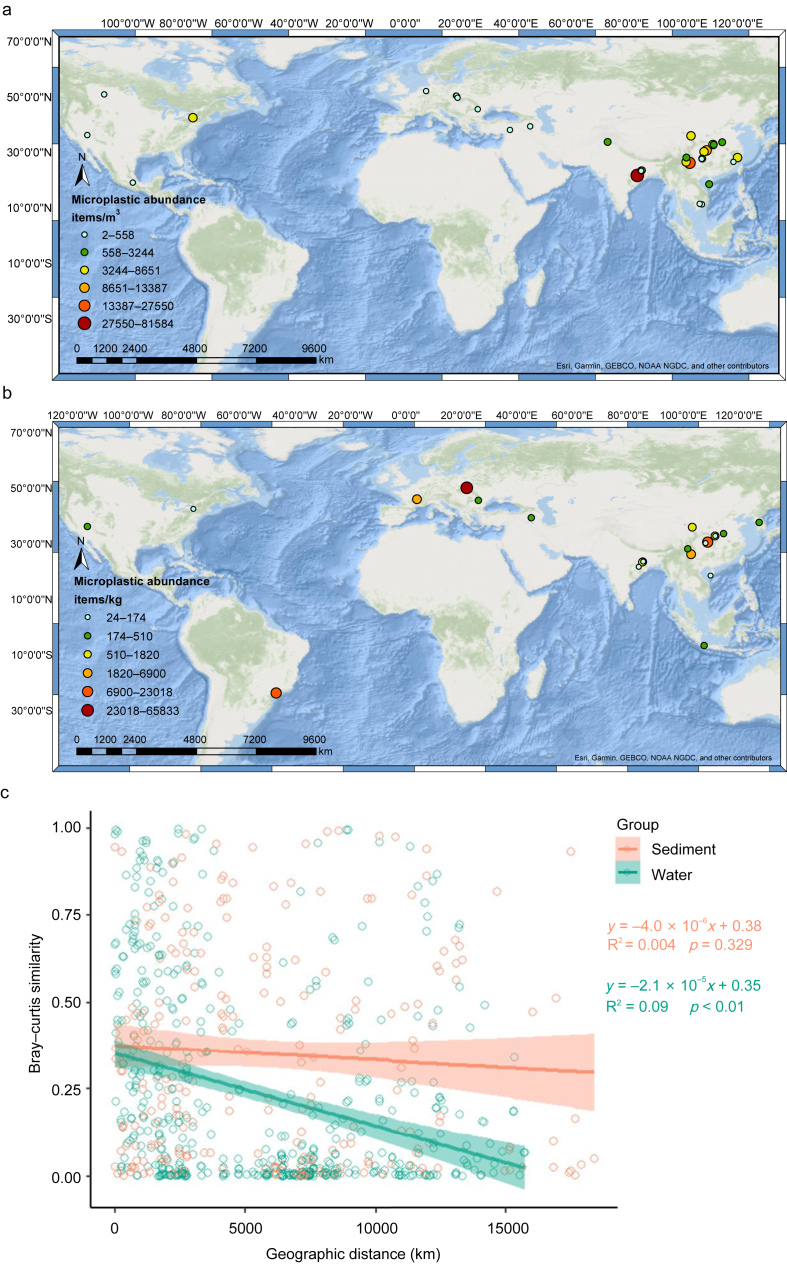
Global distribution of average MP abundance in reservoir water (a) and sediment (b). Distance–decay relationship of MP assemblage similarity in reservoir water and sediment (c).

According to the dataset, the abundance of MPs varied widely across different environments. In water, MP abundance ranged from 0.4 to 92,882.6 particles/m^3^, with a median abundance of 1717.8 particles/m^3^. The highest abundance was observed in the Hirakud Reservoir, India [40], while the lowest was found in the Hoover Dam Reservoir, USA [41]. In sediment, MP abundance ranged from 9.44 to 120,000 particles/kg with a median value of 296.4 particles/kg. The maximum concentration was recorded in the Rzeszów Reservoir, Poland [42], and the minimum in the Six-mile Reservoir, USA [21]. Fig. 1a and b present the global distribution of MP abundance in reservoirs, visualized in ArcMap. The map represents the aggregated mean values for each reservoir rather than individual sampling points, providing a broad overview of spatial variation in MP concentration. To further explore the spatial distribution patterns of MP assemblages, we examined the distance–decay relationship between geographic distance and the Bray–Curtis similarity of MP assemblage composition. As shown in Fig. 1c, a weak but significant distance–decay relationship was observed for MP assemblages in aquatic environments (n ​= ​33, slope ​= ​−2.1 ​× ​10^−5^, R^2^ ​= ​0.09, *p* ​< ​0.01), indicating that assemblage similarity decreases with increasing distance. In contrast, the distance–decay relationship for the MP assemblage in sediment was much weaker and statistically non-significant (n ​= ​22, slope ​= ​−4 ​× ​10^−6^, R^2^ ​= ​0.004, *p* ​> ​0.05), suggesting that factors other than global geographic distance, such as local hydrodynamics, sedimentation processes, and anthropogenic inputs, may play a dominant role in shaping MP assemblages in sediments.

Thirteen studies measured the abundance of MPs in both sediment and water at the same sampling sites (216 sites). Due to the non-normal distribution of the dataset, Spearman's and Kendall's correlation tests were conducted to examine the correlation between the MP abundance in water and sediment instead of using Pearson's method. In both correlation analyses, the correlation coefficient values were close to 0, and the *p*-values were greater than 0.05, indicating no significant correlation. Previous studies have similarly concluded that there is no clear relationship between MP abundance in water and sediment [[43], [44], [45]]. However, some studies suggest a correlation between MP abundance in these two compartments, although their contamination levels and ecological risk were found to be unrelated [46]. A plausible explanation for the lack of correlation is that reservoirs are dynamic systems where complex sedimentation, resuspension, and differential particle settling processes disrupt a direct relationship between the transportation of MPs in water and sediment [47,48]. Nonetheless, further research is needed to explore the potential spatial correlations between MP contamination in surface waters and sediments.

**Fig. 2:**
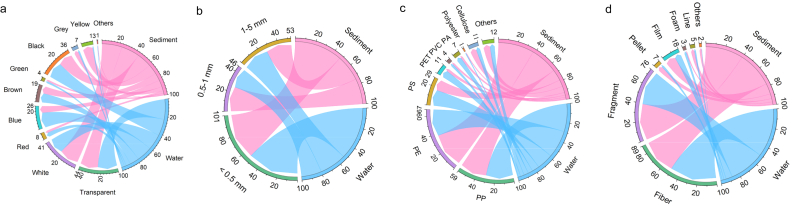
Fractions of MP characteristics (color, size, polymer composition, and shape) in water and sediment from dam–reservoir systems.

The fractions of color, shape, polymer composition, and size of MPs in dam–reservoir systems are presented in Fig. 2. In water, the most common MP colors were transparent (23.21%), black (19.18%), and white (18.23%), followed by blue (17.89%) and brown (10.72%). In sediment, white (23.16%) and transparent (21.14%) were dominant, with black (17.15%), yellow (11.70%), and blue (8.03%) being the next most abundant. MP shape fractions were similar in both compartments. In water, fragments (39.31%) and fibers (37.85%) were the most prevalent, with films (11.10%) and pellets (5.04%) also detected. In sediment, fibers (50.67%) and fragments (36.79%) were also the most common, as in water, followed by films (6.69%), foams (2.30%), and pellets (2.02%).

Polymer composition analysis revealed that PE (polyethylene) and PP (polypropylene) were the most frequently identified polymers in both water (PE: 29.03%, PP: 29.42%) and sediment (PE: 37.76%, PP:29.13%). In addition, PS (polystyrene) and PET (polyethylene terephthalate) were more prevalent in water (PS: 17.08%, PET: 7.72%), whereas PS (11.68%) and cellulose (6.65%) accounted for notable portions in sediment. Regarding MP size, particles smaller than 1 ​mm were highly prevalent, representing 73.63% of all MPs in water and 73.18% in sediment. Smaller MPs (<500 ​μm), which pose greater ecological risks, accounted for 48.01% in water and 53.10% in sediment. Overall, water has a slightly more diverse color profile, with greater proportions of black and blue MPs. The presence of cellulose in sediment and PET in water suggests varying accumulation processes between the two compartments. Despite some distinctions, MP characteristics in water and sediment showed relative similarity across the four examined properties.

### 3.2 Sources of MPs inferred from GLMM and GAMM

The global distribution of MPs in reservoirs provides an overview of large-scale patterns based on reservoir-wide averages. However, to uncover the key drivers of MP pollution, it is essential to transition from broad-scale assessments to fine-scale analyses and examine how MPs vary within individual reservoirs. To achieve this, two complementary analytical approaches were applied at different spatial scales. First, GLMM was used to assess whether MP abundance differs significantly between upstream and downstream locations of dams. In this model, the only fixed effect was upstream vs. downstream location. Second, focusing specifically on the upstream area (pre-dam reservoir), both GLMM and GAMM relationships were applied to investigate how local environmental and anthropogenic factors contribute to MP abundance at each site. This analysis incorporated multiple predictors, such as distance to the dam and population density, allowing for a more detailed understanding of the processes governing MP distribution within reservoirs.

To determine whether MP abundance differs significantly between upstream and downstream locations, the GLMM was specified as: Abundance ​∼ ​StreamPosition ​+ ​(1 | Reservoir). StreamPosition was included as a categorical predictor with two levels (Upstream and Downstream) to test whether MP abundance differed significantly along the longitudinal gradient of the reservoir. Two separate GLMMs were built for water and sediment, utilizing data from 11 studies on water sampling points and 9 studies on sediment sampling. In both models, MP abundance was the dependent variable, with upstream vs. downstream location as the fixed effect, and reservoir identifier included as a random effect to account for site-specific variability, as each reservoir has inherent characteristics and MP abundance varies widely between them.

**Fig. 3:**
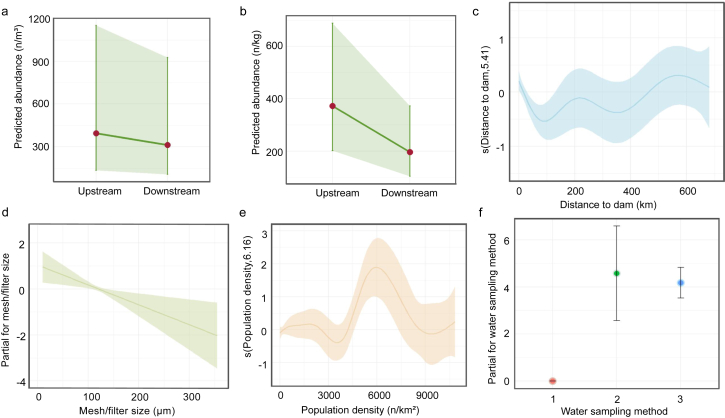
Parameter estimates for MP abundance based on GLMM and GAMM. (a) Water (GLMM), *p* ​> ​0.05; (b) Sediment (GLMM), *p* ​< ​0.05; (c) Distance to the dam (GAMM); (d) Mesh/filter size (GAMM), *p* ​< ​0.01; (e) Population density (GAMM); (f) Water sampling methods (GAMM).

Model diagnostics revealed no significant difference in MP abundance between upstream and downstream locations in water samples. However, a significant difference was observed in sediment samples. The model yielded an AIC value of 1494.0, a Bayesian Information Criterion (BIC) value of 1504.6, and a log-likelihood of −743.0. In sediment samples, the abundance of MPs in upstream locations was 89% higher than in downstream locations (*e*^0.6392^ ​= ​1.89, *p* ​< ​0.05), indicating a significantly higher upstream abundance. Model diagnostics, conducted using the DHARMa package, confirmed that the residuals from the GLMM conformed to the assumptions of normality and showed no evidence of bias or overdispersion (Fig. S1a). Additionally, the residual variance was consistent across groups, reinforcing model robustness. The findings suggest that dams primarily retain MPs in sediment rather than in water (Table S1, Fig. 3).

Meanwhile, the potential sources of MPs in the pre-dam reservoir were also evaluated. The available data for pre-dam locations were more abundant than for post-dam locations in the collected studies. The larger dataset for pre-dam locations allowed for the inclusion of additional predictors into the predictive model. These predictors were: (1) distance of the sampling site from the dam; (2) population density; (3) sampling method; (4) mesh/filter size; and (5) reservoir surface area. The predictor variable “sampling method” was categorized into three types: trawl/net sampling, in-situ filtration bulk sampling, and ex-situ filtration bulk sampling, while all other variables were treated as continuous. The reservoir identifier was still treated as a random effect, with a random intercept for each level of “Reservoir” to account for reservoir-specific variability.

Given the introduction of multiple predictors and the uncertainty regarding whether these variables have a linear relationship with the response variable, the smoothing function of the GAMM was used to capture any potential nonlinear relationships. Except for the categorical variable “sampling method”, all other variables were treated as smooth terms to account for nonlinear effects on the dependent variable. The model's AIC value was 2889.68, and the *k*-check results indicated that the *k*-indices for the smooth terms [s (mesh/filter size), s (distance to dam), s (population density), s (surface area)] were close to 1, with large *p*-values, suggesting that the smoothness selection of the model was appropriate (Table S2). The GAMM results showed that the sampling method, distance to the dam, population density, and filter size significantly influenced MP abundance (*p* ​< ​0.05) (Table S3).

Based on Fig. 3c, we observed a strong negative linear relationship between distance to the dam and MP abundance when the distance was less than 100 ​km from the dam. Additionally, most of the MP abundance data were concentrated within this range, and sampling sites located farther upstream from the dam were more likely to be less influenced by the dam's effects. Therefore, we focused on the first 100 ​km upstream of the dam for the GLMM analysis, which helped more effectively identify the linear relationships between MP abundance and various independent variables. Sites located farther than 100 ​km from the dam were excluded from the subsequent GLMM analysis.

To maintain consistency across GAMM and GLMM, the reservoir was treated as a random effect in both models. In GAMM, the only option is to include a random intercept, which accounts for variability between reservoirs but assumes the effect of the predictors is consistent across them (i.e., no varying slopes). In contrast, GLMM offers greater flexibility by allowing for both random intercepts and random slopes. To assess whether including a random slope improves the model, we compared three versions of the GLMM: (1) random intercept with a fixed slope, (2) random intercept with a random slope (correlated), and (3) random intercept with a random slope (uncorrelated). The AIC value was used to compare model performance. AIC balances model fit and complexity by penalizing the number of parameters. Lower AIC values indicate a better trade-off between goodness-of-fit and model simplicity, suggesting a more parsimonious model with less information loss. The models were ranked based on their AIC values, which were 2250.7, 2252.2, and 2252.7 for models 1, 2, and 3, respectively. Generally, a difference in AIC values of more than 2 is often considered significant, indicating that one model is meaningfully better than the other in terms of balancing goodness-of-fit and complexity. The small AIC differences between the three models indicated that introducing a random slope did not significantly improve the model. Ultimately, we selected the model formula: Abundance ​∼ ​distance_to_dam ​+ ​population_density ​+ ​sampling_methods ​+ ​mesh/filter_size ​+ ​reservoir_surface_area + (1 | Reservoir).

**Fig. 4:**
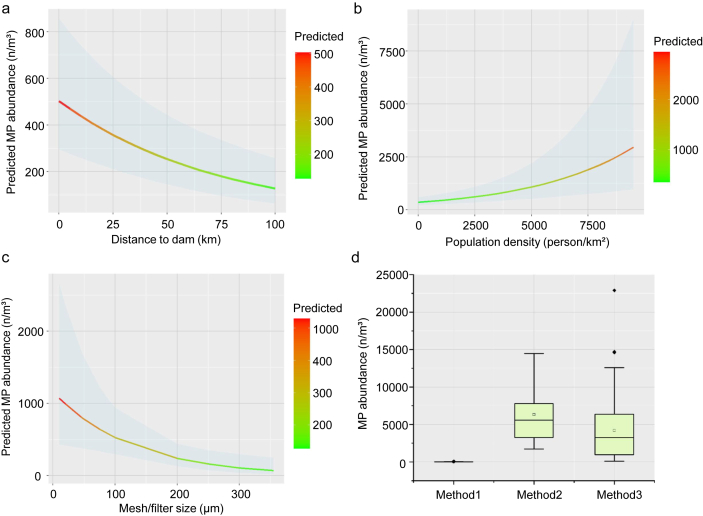
Parameter estimates for MP abundance (GLMM): (a) distance to dam (<100 ​km), *p* ​< ​0.01; (b) mesh/filter size, *p* ​< ​0.01; (c) population density; (d) water sampling methods.

The intercept is 4.091, indicating that in the absence of other influencing factors, the baseline level of MP abundance was 59.82 (after undoing the log transformation). The predicted average abundance intercept is highly significant (*p* ​< ​0.001). The coefficient for distance to the dam is −0.014, implying that for every unit increase in distance, the MP abundance decreases by 1.40% (*p* ​< ​0.001). For population density, the coefficient is 2.245 ​× ​10^−4^, suggesting that with an increase in population density, MP abundance rises by 0.25% (*p* ​< ​0.001). Regarding sampling methods, the bulk sampling methods (methods 2 and 3), compared to the baseline sampling method (method 1), both significantly increase MP abundance by 117.12 times and 69.93 times, respectively (*p* ​< ​0.001 for both). The coefficient for mesh/filter size is −0.0079, meaning that for each unit increase in available size, MP abundance decreased by 0.79% (*p* ​< ​0.05). For reservoir surface area, the coefficient indicates that with each unit increase, MP abundance increases by 0.072%. However, the surface area effect is not statistically significant (*p* ​> ​0.05), indicating its minimal impact on the model (Fig. 4, Table 1). Model diagnostics for this model also showed no evidence of bias or overdispersion (Fig. S1b).

**Table 1:** Significant effects of multiple predictors on MP abundance in pre-dam reservoir water (GLMM).

	Estimate	Std. Error	value	Pr (>|z|)	Sign. code
(Intercept)	4.091	0.694	5.894	3.77 ​× ​10^−9^	∗∗∗
Distance to dam	−0.014	2.83 ​× ​10^−3^	−4.831	1.36 ​× ​10^−6^	∗∗∗
Population density	2.245 ​× ​10^−4^	5.52 ​× ​10^−5^	4.066	4.79 ​× ​10^−5^	∗∗∗
Sampling method 2	4.763	0.911	5.228	1.72 ​× ​10^−7^	∗∗∗
Sampling method 3	4.247	0.314	13.528	<2 ​× ​10^−16^	∗∗∗
Mesh/filter size	−0.008	2.79 ​× ​10^−3^	−2.833	0.00462	∗∗
Surface area	7.192 ​× ​10^−4^	6.93 ​× ​10^−4^	1.039	0.29899	

Significance codes: ∗∗*p* ​< ​0.01; ∗∗∗*p* ​< ​0.001.

In summary, increasing the variable value “distance to dam” and “mesh/filter size” may lead to a slight decrease in MP abundance. Population density had a small but positive effect on MP abundance. Water sampling methods significantly influence the estimates of abundance, with the bulk sampling method substantially increasing the detected number of MPs. Building on these findings for water, we also attempted to model MPs in pre-dam sediment using both GLMM and GAMM approaches. However, due to the limited variety in sediment sampling methods (primarily grab sampling), we excluded the sampling method as a predictor in the models. Despite this, both models performed poorly, and no significant relationships were found between the explanatory variables and MP abundance in sediment (*p* ​> ​0.05). This suggests that the factors influencing MP distribution in sediment may be more complex or less directly related to the variables used in our models, compared to the clearer patterns observed in water. Significant differences in the distribution of MPs between water and sediment in reservoirs were observed. Firstly, the distance–decay relationship showed a linear decrease in MP abundance similarity with increasing geographic distance between reservoirs for water samples, indicating that the MPs in water were strongly influenced by regional and spatial factors. In contrast, MPs in sediment did not exhibit this distance–decay relationship, suggesting that local environmental factors, such as sedimentation, resuspension, and hydrodynamics, play a larger role in determining their distribution.

Secondly, the effect of dams differs between water and sediment. In sediment, MP abundance was significantly higher upstream of the dam than downstream (*p* ​< ​0.05), reflecting a pronounced trapping effect of the dam on sediment-bound MPs. However, in water, there was no significant difference in MP abundance between upstream and downstream (*p* ​> ​0.05). This could be due to the more dynamic nature of MPs in water, where short-term variations such as rapid horizontal transport during dam operations and high-flow periods in rainfall seasons might mask the dam's effect on MP distribution.

Finally, in the pre-dam reservoir region (within 100 ​km of the dam), GLMM indicated significant linear relationships between MP abundance in water and variables like mesh size and distance to the dam. For example, larger mesh sizes captured fewer MPs, and sites closer to the dam had higher MP concentrations, suggesting that the dams influence MP abundance in water. However, in sediment, no significant linear relationships were found, indicating that the distribution of sediment-bound MPs is governed by more complex local sedimentary processes, which cannot be easily captured by linear models.

In summary, both water and sediment MPs are affected by the dam's trapping effect but exhibit distinct distribution patterns. MPs in water are primarily governed by regional transport (e.g., hydrological flow and pollution source dispersion), showing spatial continuity. In contrast, sediment MPs are dominated by settling and resuspension, leading to stronger spatial heterogeneity. Dams significantly trap MPs in sediments, with upstream abundance 89% higher than downstream. This pattern can be explained by well-established hydrodynamic mechanisms. Dams reduce flow velocity and increase water residence time in the upstream zone, creating low-energy environments that promote the settling of MP particles. Although flow velocity was not directly modeled in this study, spatial variables such as upstream/downstream location and distance to the dam effectively capture the hydrodynamic gradient. This highlights the possibility of near-dam targeted dredging to remove MPs. However, heavy floods or storms might resuspend trapped MPs and redistribute them. In cold regions, MPs may undergo freezing and thawing, re-entering the water column [49]. For MPs in water, their constant movement means that pollution mitigation requires coordinated efforts across the entire river basin to control sources like wastewater or plastic waste. Since the GLMM/GAMM struggled to identify key drivers of MPs in sediment, future work could combine sediment-hydrodynamic models with high-resolution monitoring to better understand complex sediment transport processes.

### 3.3 Vertical distribution of MPs in reservoirs

**Fig. 5:**
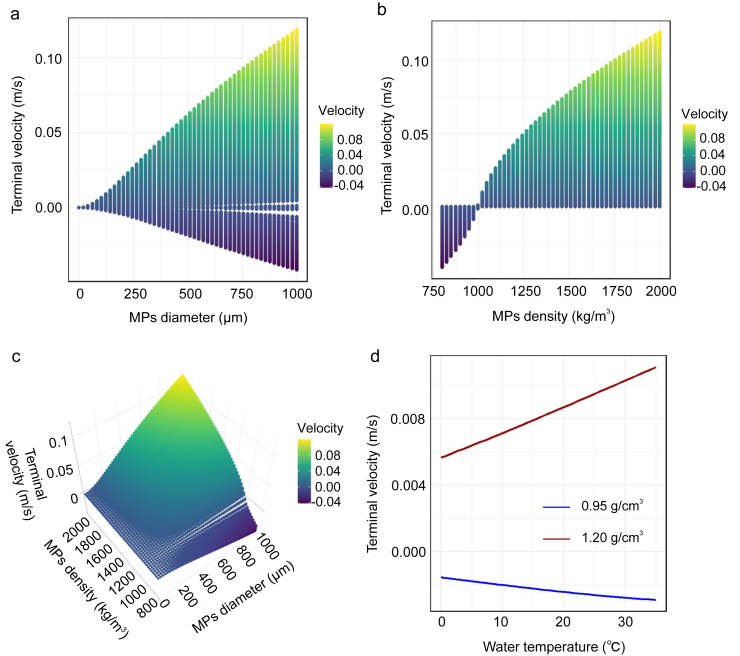
Relationship between MP diameter and terminal velocity (a), relationship between MP density and terminal velocity (b), three-dimensional surface plots of MP density, diameter and terminal velocity (c), the effect of water temperature on terminal velocity, with *d* set to 333 ​μm and *ρ*_*p*_ set to 950 and 1200 ​kg/m^3^, respectively (d).

Based on the theoretical model described in *Section* 2.3, we calculated the vertical settling velocity of MPs under varying conditions. For Fig. 5d, with *d* set to 333 ​μm and *ρ*_*p*_ set to 950 and 1200 ​kg/m^3^, respectively, the relationship between water temperature and terminal velocity was plotted. As shown in Fig. 5, as the density and diameter of MPs increase, so does their terminal velocity, leading to faster sinking. While the theoretical calculations predict that common low-density polymers like PP and PE should float, our previous statistical data have revealed that over 66.89% of MPs in reservoirs consist of these low-density types. This paradox is explained by several key mechanisms. Biofilm formation plays a critical role, as microbial colonization increases the effective density and size of MPs, facilitating their sinking [50,51]. The aggregation of MPs with iron colloids and microbes within biofilms further increases their settling velocity [52]. Additionally, hydrodynamic forces, such as turbulence and density currents, can transport buoyant MPs to deeper water layers, where thermal stratification may hinder their upward movement. Ecological interactions, including plankton ingestion and benthic organisms' bioturbation, also contribute significantly to the vertical transport of MPs. For instance, when zooplankton ingest MPs, they produce fecal pellets, which facilitate the vertical transportation of floating plastics from surface water [53]. Similarly, benthic organisms may bury MPs in sediments through their burrowing behavior [54].

The vertical distribution of MPs in reservoirs exhibits significant variability across studies, reflecting the complexity of governing mechanisms. Zhang et al. [5] observed higher MP concentrations in the hypolimnion (the deepest layer according to the thermal stratification) due to elevated iron and manganese levels, which promote colloid formation and MP aggregation, facilitating their sinking. Biofilm formation further enhances this process. Conversely, Liu et al. [55] reported surface MP enrichment linked to algal aggregation, while Lin ​et al. [56] identified peak concentrations in intermediate layers, where stagnant water flow likely contributes to MP accumulation. Even sediment core analyses show contradictions: Baldwin et al. [41] found no clear depth trend despite peak MPs in the deepest samples.

Particle size distributions further complicate this picture. Liu et al. [55] documented an increase in large MPs (1–5 ​mm) and a decline in small MPs (75–200 ​μm) with depth, contrasting with Lin's [56] observation of a higher percentage of fine particles (<0.2 ​mm) in deeper layers. Zhang et al. [5], however, found no systematic vertical size variations.

This evidence underscores the multifaceted controls on MP transport in reservoirs: competing effects of biofilms (enhanced settling) vs. turbulent mixing (prolonged suspension), density stratification barriers, and site-specific geochemical conditions [57,58]. Inconsistent patterns across research indicate the need for further study. The limitation of conventional models (GLMM and GAMM) to establish universal patterns highlights the need for future frameworks integrating hydrodynamic, biogeochemical, and ecological drivers across spatiotemporal scales.

### 3.4 Trapping effects of river dams and cascade reservoirs on MPs

Dams, as critical infrastructures altering riverine ecosystems, create reservoirs by impeding natural water flow. Although many studies on dam–river interactions did not explicitly define reservoirs, their findings remain relevant to reservoir systems due to shared mechanisms in sediment and pollutant retention. It is estimated that over 62,000 large dams exist globally [59], many of which significantly affect river dynamics. Worldwide, more than 1.6 million km of rivers have lost their free-flowing status, with only one-third of rivers longer than 1000 ​km remaining free-flowing [60]. This widespread disruption necessitates evaluating the role of both single river dams and cascade reservoirs—sequential dams that amplify or modulate impacts through cumulative interactions.

Emerging studies suggest dams may act as MP sinks, though their efficacy varies significantly. Lebreton et al. [61] demonstrated that artificial barriers (including dams) could retain up to 65% of MPs entering freshwater systems. By correlating mismanaged plastic waste (MPW) with downstream MP concentrations, they found that considering dam retention significantly improved the correlation (*r* increased from 0.17 to 0.41), implying that dams amplify the linkage between terrestrial plastic pollution and aquatic MP loads. However, dam-induced MP trapping is not universally observed. In the Orange-Vaal River system, dams showed minimal retention of buoyant MPs [62]. Conversely, within the same basin, Graham et al. [63] reported microlitter concentrations in the Bloemhof Dam 6- to 70-fold higher than at other sites. This contrast highlights the role of local drivers, such as MP properties, dam operation regimes, and hydraulic conditions.

Meanwhile, cascade reservoirs introduce additional complexity to MP dynamics. A series of dams along a river creates “staircase” reservoirs. Studies in the Shaying River Basin revealed that MP abundance upstream of each individual dam was consistently higher than downstream, suggesting short-term retention within reservoirs [64]. Similar patterns were observed in the Fall Creek dams and Six Mile Creek dams, where MP concentrations decreased immediately downstream of each dam [21]. These findings support the hypothesis of a “filtering cascade” effect, where sequential dams progressively reduce MP flux toward estuaries. However, the hypothesis often fails to hold in real-world observations. Whole-river assessments typically reveal an overall increase in MPs from headwaters to downstream. In the Wujiang River, for example, MP concentrations in sediments from the nine cascade reservoirs along the river gradually increased, from 310 to 1670 items/kg dw [65]. The paradox between localized retention versus basin-wide accumulation is further corroborated by Watkins et al. [21], who found no cumulative reduction in MP concentrations across the dam series despite high retention efficiencies at individual dams. This discrepancy can be attributed to several interconnected mechanisms. Tributaries and urban/agricultural runoff between dams continuously introduce new MPs, offsetting retention effects, while coordinated water releases for hydropower generation or flood control may flush MPs from upstream reservoirs into lower reaches. Additionally, MPs trapped in upstream reservoirs may fragment or degrade into smaller, more mobile particles that evade subsequent retention. In summary, while cascade dams temporarily buffer MP transport, they may inadvertently create “accumulation hotspots” in midstream and downstream reservoirs.

### 3.5 Ecological impacts of MPs in reservoirs: a focus on benthic organisms

Previous analyses have demonstrated that MPs in reservoirs tend to accumulate near dams, with a pronounced effect on sediment layers compared to the water column. This “dam-centric” accumulation pattern raises critical ecological concerns, especially for benthic organisms, the primary inhabitants of sediment–water interfaces directly exposed to elevated MP loads [66]. Benthic organisms play vital roles in reservoir ecosystems, mediating biogeochemical cycles (e.g., organic matter decomposition and nutrient recycling) and serving as keystone species in aquatic food webs [67]. However, exposure to high concentrations of MPs may render them vulnerable to both physical (e.g., abrasion, gut blockage) [68] and chemical (e.g., leached additives, adsorbed pollutants) [69] MP-related stressors. Furthermore, the long-term burial of MPs in sediments suggests chronic exposure risks, potentially disrupting benthic population dynamics and higher trophic levels [70].

**Fig. 6:**
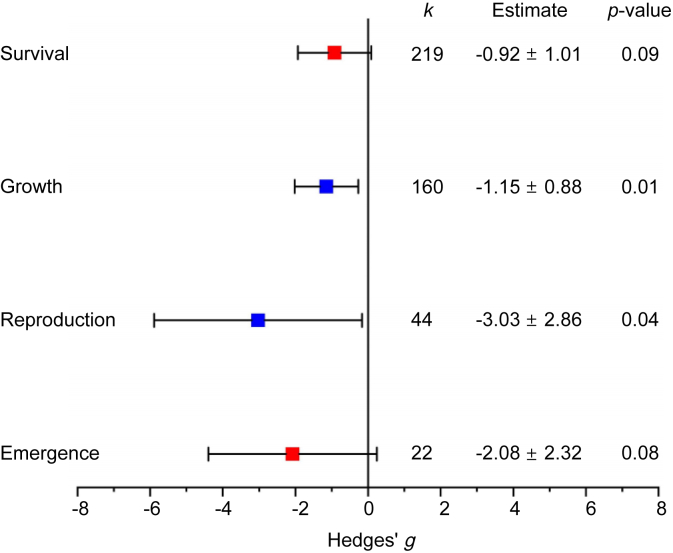
Effect of MPs on the functional traits of benthic organisms. Analysis conducted with mixed effects model, using the rma.mv function of the metafor package in R, including study ID as a random factor. Boxes represent Hedges' *g* value, and the horizontal lines represent the 95% CI for each g value.

To quantitatively assess the ecological impacts of MPs on benthic organisms in reservoir ecosystems, we conducted a meta-analysis that included four biological response categories: survival, growth (length and weight), reproduction, and emergence. While the included studies span a range of freshwater systems, many of the benthic taxa used in experimental studies, such as oligochaetes, chironomids, and amphipods, are lentic-adapted species known to tolerate the low-oxygen, fine-sediment conditions prevalent in reservoirs. Thus, the effects observed in this meta-analysis are ecologically meaningful for species likely to dominate reservoir benthic communities. The estimated effect size (Hedges' *g*) and its 95% confidence intervals (CIs) are summarized in Fig. 6.

Growth showed a significant negative effect (Hedges' *g* ​= ​−1.15, *p* ​< ​0.01), indicating that MPs significantly reduce the growth of benthic organisms. Reproduction was also significantly affected (Hedges' *g* ​= ​−3.03, *p* ​< ​0.05), suggesting a strong negative impact of MPs on reproductive success. The other two functional traits, survival and emergence, showed negative trends, but the effects were not statistically significant at the conventional *α* ​= ​0.05 level. This discrepancy might be explained by inherent differences in the sensitivity of functional trait responses, the heterogeneity of experimental designs, compensatory mechanisms at the organismal level, and potential long-term cumulative effects.

Growth and reproduction are sublethal endpoints that can reflect physiological stress and energy allocation shifts more directly and quickly than survival. MP exposure might affect energy budgeting, nutrient absorption, or endocrine regulation, leading first to reduced growth and reproduction. In contrast, changes in survival might only be evident at higher concentrations or after prolonged exposure. Moreover, organisms might employ compensatory strategies when facing environmental stress (e.g., reducing growth to prioritize survival). As a result, the immediate impact is observed in growth and reproduction, while survival rates and emergence might remain relatively unchanged in the short term or require longer observation periods to manifest significant changes.

To further support these conclusions, we conducted subgroup analyses for survival and growth using exposure time as a covariate, while excluding emergence and reproduction due to limited sample sizes. Subgroup analyses also support these findings (Fig. S2). For survival, effect size values were −0.16 (*p* ​> ​0.05) for exposures <10 days, −0.38 (*p* ​< ​0.01) for 10–25 days, and −1.34 (*p* ​< ​0.01) for >25 days. Similarly, growth showed an effect size of −0.16 (*p* ​> ​0.05) for exposures <25 days and −2.07 (*p* ​< ​0.01) for exposures >25 days. These findings further verify that prolonged MP exposure has increasingly detrimental effects on both survival and growth. We did not conduct subgroup analyses based on MP concentration or particle type due to inconsistent units (e.g., particle/mL vs. g/mL) and the wide variation in particle sizes reported across studies, which makes cross-study standardization infeasible. Likewise, subgroup analyses based on benthic traits (e.g., filter feeders vs. deposit feeders) or MP forms (e.g., fibers vs. fragments) were not possible due to insufficient or inconsistent reporting.

Several studies report the frequent co-occurrence of non-significant survival/mortality outcomes with significant growth or reproductive impairments [71,72], suggesting that the MP concentrations used (simulation of natural environmental MP concentration) are generally below acute toxicity thresholds for benthic organisms. Future experiments should consider focusing on acute (e.g., <48 ​h) exposures at higher MP concentrations to more effectively evaluate survival outcomes. Even if survival and emergence do not show significant effects in the short term, sublethal effects on growth and reproduction can reduce recruitment and destabilize populations over time. Moreover, as MPs frequently co-occur with other contaminants, cumulative impacts may be underestimated. Long-term, multi-generational experiments incorporating multiple stressors are needed to better capture the ecological consequences of chronic MP exposure.

### 3.6 Evolving policies and challenges in global MP governance

Compared to secondary MPs, which are harder to define, the first campaigns raising awareness about MPs specifically targeted primary MPs [73]. These include those intentionally added to products, such as microbeads in cosmetic and personal care products. One of the earliest legislative actions was the Microbead-Free Waters Act of 2015 in the United States, which prohibited the manufacture and sale of rinse-off cosmetics containing microbeads [74]. Following this, many developed countries introduced similar regulations. Canada's Microbeads in Toiletries Regulations and the U.K. Environmental Protection (Microbeads) Regulation 2017 prohibited the manufacture and sale of microbead-containing products [75,76] French Decree 2017-291 imposed similar restrictions [77].

Beyond cosmetics, a few regulations have addressed MPs in other product types. The EU's REACH (Registration, Evaluation, Authorization, and Restriction of Chemicals) Regulation (2023/2055) restricts synthetic polymer microparticles under Annex XVII, prohibiting MPs ≥ 0.01% by weight in substances or mixtures, with specific exemptions for industrial use [78].

At the same time, regulatory efforts targeting secondary MPs are gaining momentum. For instance, France's Anti-Waste and Circular Economy Law (AGEC) mandates that all new washing machines sold from 2025 must include microfiber filters [79]. Similarly, California Assembly Bill 1628 requires all washing machines sold by 2029 to be equipped with microfiber filtration systems [80].

In addition to mandatory laws, voluntary guidelines and recommendations have emerged. Samsung Electronics has implemented internal guidelines encouraging microfiber filter installation [81], while Australia's National Plastics Plan promotes microfiber capture technologies and advocates for phasing out oxo-degradable plastics [82]. The Swedish Textile Initiative for Climate Action guides manufacturers to minimize microfiber release during production and consumer use [83].

**Fig. 7:**
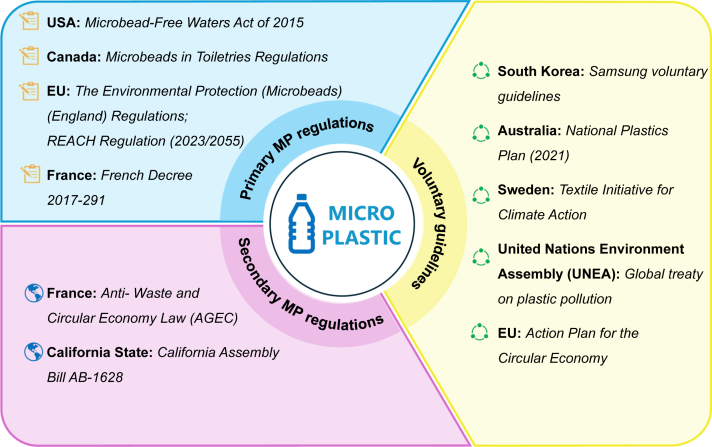
Global regulatory and voluntary frameworks for MP pollution management.

Despite the progress made, many proposed policies remain under discussion. The United Nations Environment Assembly (UNEA) is currently negotiating a Global Treaty on Plastic Pollution, aiming to address both primary and secondary MPs by 2025 [84]. The EU Circular Economy Action Plan further proposes additional restrictions on MP emissions from textiles, tire wear, and plastic pellet losses during production [85]. The U.K. is also reviewing a proposal to mandate microfiber filters in washing machines, aligning with regulations in France and California (Fig. 7).

However, current global MP governance strategies rarely address the unique challenges posed by dam–reservoir systems. Our study shows that MPs tend to accumulate near dams, particularly in sediments, leading to chronic ecological risks to benthic organisms. Given these findings, future policies should explicitly consider the lentic and depositional nature of reservoirs, which distinguishes them from more dynamic river systems. This could involve incorporating MP monitoring into existing sediment management and dam operation protocols, particularly in zones prone to MP retention. Additionally, upstream source control should be strengthened by enhancing solid waste management and wastewater treatment infrastructure in reservoir catchments, thereby reducing the influx of MPs before they accumulate. Reservoir-specific regulations can help mitigate MP accumulation and safeguard aquatic biodiversity.

Finally, while promoting global MP regulations, the risk of greenwashing must be underlined [86]. Some enterprises exaggerate their environmental contributions or use ambiguous claims, such as “microplastic-free” labels or ill-defined recycling programs. To ensure effective measures against MP pollution, regulatory frameworks must emphasize transparency, independent oversight, and evidence-based evaluations to avoid misleading practices that delay effective action.

## 4 Conclusions

This study provides new insights into the distribution of MPs in reservoirs, highlighting the role of dams in MP accumulation and transport. The observed accumulation of MPs near dams, coupled with declining downstream abundances, underscores the trapping effect of dam–reservoir systems, which can significantly alter the transport dynamics of MPs in water and sediment. The vertical stratification patterns further reveal the complexity of MP behavior in reservoirs, suggesting that hydrodynamic processes, sediment interactions, and water column dynamics play critical roles in MP distribution. Additionally, the adverse effects of MP exposure on benthic organisms, particularly in terms of growth and reproduction, raise significant ecological concerns. Prolonged exposure exacerbates these impacts, indicating that reservoirs may act as long-term sinks for MPs, with potential effects on aquatic food webs and ecosystem health.

By addressing these challenges, we can better understand and mitigate the ecological risks posed by MPs in freshwater systems, ensuring the sustainability of reservoir ecosystems and the services they provide. Future research should focus on elucidating the mechanisms driving MP accumulation and transport in reservoirs, including the role of sediment dynamics, hydrological conditions, and dam operations. Long-term studies are needed to assess the cumulative ecological impacts of MPs on benthic communities, as well as their potential transfer to higher trophic levels. Policymakers and water resource managers should prioritize the development of integrated strategies to mitigate MP pollution in reservoirs, including improved waste management, upstream pollution control, and the implementation of advanced filtration technologies at dam sites.

## CRediT authorship contribution statement

**Wei Gao:** Writing – original draft, Visualization, Validation, Methodology, Investigation, Formal analysis, Data curation, Conceptualization. **Peng Zhang:** Writing – review & editing. **Hongcui Wang:** Writing – review & editing. **Xiaohan Yang:** Data curation. **Chunjiang An:** Writing – review & editing, Conceptualization.

## Declaration of competing interests

The authors declare that they have no known competing financial interests or personal relationships that could have appeared to influence the work reported in this paper.
