# Supplementary material for: From water to sediment: A meta-analysis of microplastic distribution and the impact of dams in reservoir ecosystems
Source: Eco Environ Health. 2025 Sep 26;4(4):100188. doi: 10.1016/j.eehl.2025.100188 (PMC12596587; doi:10.1016/j.eehl.2025.100188)
Supplement: Multimedia component 1 [file mmc1.pdf]

# SUPPLEMENTARY MATERIAL

## From Water to Sediment: A Meta-analysis of Microplastic Distribution and the Impact of Dams in Reservoir Ecosystems

Wei Gao [a, b*], Peng Zhang [c], Hongcui Wang [b], Xiaohan Yang [a], Chunjiang An [a*]

[a] Department of Building, Civil and Environmental Engineering, Concordia University, Montreal, Quebec H3G 1M8, Canada

[b] Center of Eco-environmental Monitoring and Scientific Research, Administration of Ecology and Environment of Haihe River Basin and Beihai Sea Area, Ministry of Ecology and Environment of the People's Republic of China, Tianjin 300211, China

[c] Institute for Energy, Environment and Sustainable Communities, University of Regina, Regina, Saskatchewan S4S 0A2, Canada

*Corresponding Authors:

Wei Gao − Center of Eco-environmental Monitoring and Scientific Research, Administration of Ecology and Environment of Haihe River Basin and Beihai Sea Area, Ministry of Ecology and Environment of the People's Republic of China, Tianjin 300211, China; Email: gaowei@hhbhjg.mee.gov.cn.

Chunjiang An − Department of Building, Civil and Environmental Engineering, Concordia University, Montreal, Quebec H3G 1M8, Canada; Phone: 1-514-848-2424 ext. 7857; Email: chunjiang.an@concordia.ca.

## Content

Supplementary text: Methods for meta-analysis of benthic organisms

Table S1 – S4

Figure S1 – S2

Appendix S1 – S2

References list

## Methods for Meta-Analysis of Benthic Organisms

### Literature Search and Inclusion Criteria

A systematic literature search was conducted on Web of Science to identify studies examining the effects of microplastics on benthic organisms in December 2024 (Moher et al., 2009; Pullin and Stewart, 2006). The search was performed using the following search string: ("microplastic" OR "microplastics") AND (benthic*) AND (freshwater). The initial database search yielded 118 studies, which were screened according to the following criteria: (1) The study investigated the effects of microplastics on benthic organisms in freshwater; (2) The study used a controlled experimental design, with at least one control group (Ck) and one treatment group (Tr); (3) The study focused on functional traits at the individual level rather than population-level effects and reported at least one of the following response variables: survival or mortality rate, growth (weight or length), reproduction, or emergence; (4) To avoid confounding effects of food availability on MP ingestion, studies were excluded if they did not provide food or treated food as a control variable; (5) The study examined the single-factor effects of MPs, meaning that studies where MPs were not the sole experimental factor were excluded; (6) Sufficient statistical data (mean, standard deviation or standard error, and sample size) were provided or could be extracted from figures; (7) The review articles, meta-analyses, or theoretical studies were excluded. After this screening process, 19 studies were included in the meta-analysis (Appendix S2).

**Data Extraction**

For each eligible study, the following data were extracted: (1) Study characteristics: Authors, publication years, experimental conditions (e.g., MP type, concentration, exposure duration); (2) Biological information: Species name and developmental stage (larvae or adult); (3) Response variables: Survival rate, growth, reproduction, emergence (Violle et al., 2007); (4) Statistical data: Mean ($\bar{X}$), standard deviation (SD) or standard error (SE), and sample size (n) for both control and treatment groups.

**Effect Size Calculation**

To quantify the effect of microplastics on freshwater benthic organisms, we calculated standardized effect sizes using Hedges'g (Gurevitch and Hedges, 2020; Hedges, 1981), which accounts for small sample bias. The effect size for each study was computed as follows:

$$g = \frac{X_{Tr} - X_{ck}}{S_{pooled}} \times J$$

where $X_{Tr}$ and $X_{ck}$ are the mean of the experimental treatment and control groups respectively, $S_{pooled}$ is the pooled standard deviation, and $J$ corrects for small sample bias (Borenstein et al., 2021; Koricheva et al., 2013).

$$J = 1 - \frac{3}{4(N_{Tr} - N_{ck} - 2) - 1}$$

$$S_{pooled} = \sqrt{\frac{(N_{Tr} - 1) \times SD_t^2 + (N_{ck} - 1) \times SD_c^2}{N_{Tr} + N_{ck} - 2}}$$

where $N$ is the sample size and SD is the standard deviation of the treated or control group. The Shapiro-Wilk test of the effect sizes rejected normality. Given the pronounced skewness and kurtosis, we used non-parametric methods to calculate the standard deviation, which didn't rely on the normal distribution of size effect (De Graaff

et al., 2006).

$$V_g = \frac{N_t + N_c}{Nt \times N_C}$$

As the sign of Hedges' g tells the direction of the effect, a negative value of Hedges' g indicates that microplastics have a higher effect on impairing that specific analyzed response.

**Statistical Analysis**

In the step of calculating the total Hedges' g value, a multilevel random effects model was used to account for variability across studies. The analysis was conducted using the rma.mv function from the R package metafor (Viechtbauer, 2010). Study_ID was included as a random effect to account for non-independence among effect sizes from the same study (Viechtbauer, 2007). Effect sizes were considered to be significant if their 95% confidence interval (CI) did not overlap with zero and if their $p<0.05$. Differences between the groups included as moderators in the subgroup analysis were considered to be significant when the p-value of the test for moderators (Qm) calculated in the mixed effects model was $< 0.05$.

**Subgroup Analysis**

We initially examined multiple moderators (e.g., species, MP type, exposure concentration), but only exposure duration showed meaningful subgroup differences. Other subgroup analyses were excluded due to non-significant or biologically inconsistent patterns. Among the four functional traits, only survival and growth were analyzed in subgroups, as sample sizes for the other traits were insufficient for meaningful subgroup analysis (typically requiring a minimum of 10 samples per group from 3 individual literatures).

**Publication Bias Assessment**

Publication bias was assessed using Egger's regression, which regresses

standardized effect sizes on the logarithm of their standard errors to correct for skewed SE distributions (Egger et al., 1997). Given that our nonparametric variance estimator may compromise funnel plot symmetry, we prioritized Egger's regression as the primary bias assessment tool. Across all response categories, the intercepts were non-significant ($p > 0.05$, Table S4), indicating no detectable publication bias. Although funnel plots are commonly used for visualizing bias, any asymmetry observed is likely due to our conservative variance method rather than true bias.

**Table S1.** Significant effects of upstream-downstream difference on MP abundance in reservoir sediment and water (GLMM). Significance codes: * $p < 0.05$; ** $p < 0.01$; *** $p < 0.001$.

| | Estimate | Std. Error | z value | Pr (> \|z\|) | Sign. code |
|---|---|---|---|---|---|
| **Intercept** | 5.281 | 0.326 | 16.213 | < 2e-16 | *** |
| **Downstream (sediment)** | 0.639 | 0.150 | 4.259 | 2.05e-05 | *** |
| **Intercept** | 5.745 | 0.554 | 10.364 | < 2e-16 | *** |
| **Downstream (water)** | 0.230 | 0.145 | 1.589 | 0.112 | |

**Table S2.** Post-fitting test statistics for GAMM smooth terms.

| | k | edf | k-index | p-value |
|---|---|---|---|---|
| **s(FZ)** | 9 | 1.002 | 0.920 | 0.203 |
| **s(Dis)** | 9 | 5.308 | 0.985 | 0.553 |
| **s(population_density)** | 9 | 6.138 | 1.055 | 0.890 |
| **s(surface_area)** | 9 | 1.000 | 0.947 | 0.335 |
| **s(Site)** | 18 | 13.189 | NA | NA |

**Table S3.** Summary of estimated coefficients and smooth terms in the GAMM

| Predictor/smooth term | Type | Estimate/edf | Std. Error/Ref. df | z/Chi.sq | p-value |
|---|---|---|---|---|---|
| **(Intercept)** | Parametric | 3.399 | 0.419 | 8.120 | 4.65e-16 |

| | | | | | |
|---|---|---|---|---|---|
| **WSM2** | Parametric | 4.578 | 1.006 | 4.551 | 5.34e-06 |
| **WSM3** | Parametric | 4.174 | 0.328 | 12.745 | 3.31e-37 |
| **s(FZ)** | Smooth | 1.002 | 1.002 | 7.773 | 5.35e-03 |
| **s(Dis)** | Smooth | 5.308 | 6.396 | 20.723 | 2.87e-03 |
| **s(population_density)** | Smooth | 6.138 | 7.170 | 22.464 | 2.60e-03 |
| **s(surface_area)** | Smooth | 1.000 | 1.000 | 0.876 | 3.49e-01 |
| **s(Site)** | Random smooth | 13.189 | 15.000 | 385.428 | <0.001 |

**Table S4.** Random-effects model effect size estimates and assessment of potential publication bias.

| **Effect size metric** | **Random-effects model** | | | **Potential publication bias** | |
|---|---|---|---|---|---|
| | ***n*** | ***Hedge's g*** | ***± 95% CI*** | ***t-value*** | ***p-value*** |
| **Survival** | 219 | -0.918 | 1.012 | -1.680 | 0.094 |
| **Growth** | 160 | -1.145 | 0.876 | 0.271 | 0.787 |
| **Reproduction** | 44 | -3.029 | 2.863 | -1.039 | 0.311 |
| **Emergence** | 22 | -2.083 | 2.323 | 0.233 | 0.817 |

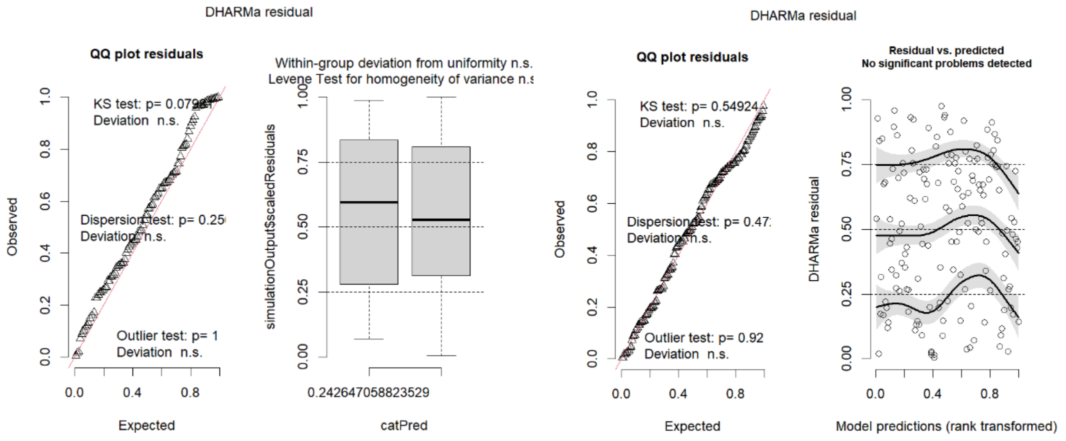


**Fig. S1.** Residual diagnostics for hierarchical mixed regression models (a) upstream-downstream on microplastic abundance in reservoir sediment (b) multi-predicted variables on microplastic abundance in pre-dam reservoir water.

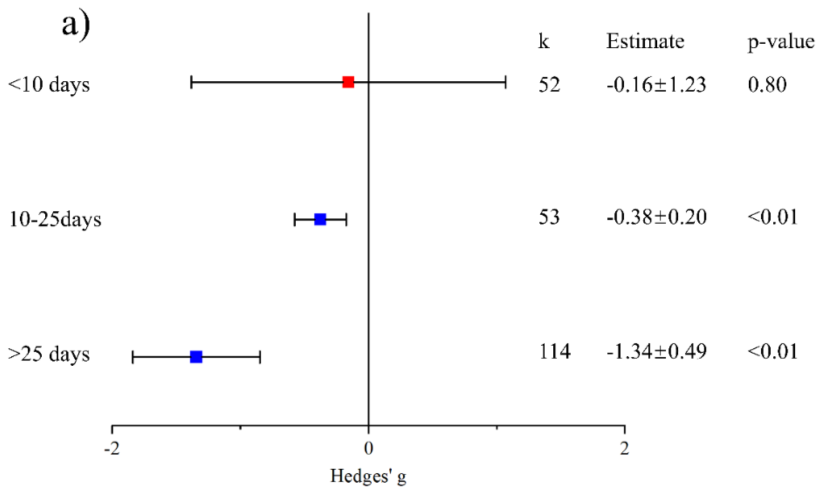


Test of Moderator (coefficient: exposure duration)

| | Qm | df | p-val |
|---|---|---|---|
| Between groups | 32.39 | 2 | <0.01 |

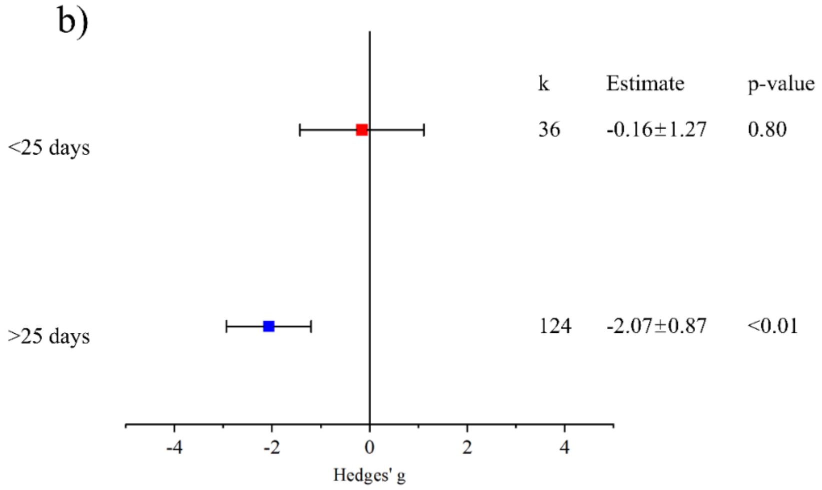


Test of Moderator (coefficient: exposure duration)

| | Qm | df | p-val |
|---|---|---|---|
| Between groups | 21.78 | 1 | <0.01 |

**Fig. S2.** Forest plot of the "exposure duration" subgroups effect-size. Analysis conducted with mixed-effects model, using the rma.mv function of the metaphor package in T, including Study_ID as random factor. Black boxes represent the Hedges'g value and the horizontal lines represent the 95% CI for each g value; Qm =omnibus test of moderators from the model; k = number of study cases.

**Appendix S1. Bibliography of studies included in the meta-analysis of microplastics distribution in dam-reservoir.**

| Authors | Title | Year | Journal | Reservoir |
|---|---|---|---|---|
| Nocoń et.al. | Microplastics upstream and downstream dam-reservoirs | 2020 | Desalination and Water Treatment | Pławniowice dam-reservoir; Dzierżno Małe dam-reservoir; Łąka dam-reservoir |
| Lin et.al. | Distribution and source of microplastics in China's second largest reservoir - Danjiangkou Reservoir | 2021 | Journal of Environmental Sciences | Danjiangkou reservoir |
| Patra et.al. | Assessment of microplastics and associated ecological risk in the Hirakud Reservoir, Odisha, India | 2024 | Journal of Water and Health | Hirakud reservoir |
| Niu et. al. | Occurrence, stability and source identification of small size microplastics in the Jiayan reservoir, China | 2022 | Science of the Total Environment | Jiayan reservoir |

| | | | | |
|---|---|---|---|---|
| Liu et.al. | Horizontal and vertical distribution of microplastics in dam reservoir after impoundment | 2022 | Science of the Total Environment | Guanyinyan reservoir |
| Xu et.al. | Influence of catastrophic flood on microplastics organization in surface water of the Three Gorges Reservoir, China | 2022 | Water Research | Three Gorges dam reservoir |
| Gao et.al. | Substantial burial of terrestrial microplastics in the Three Gorges Reservoir, China | 2023 | Communications Earth & Environment | Three Gorges dam reservoir |
| Min et.al. | Distribution and risk assessment of microplastics in Liujiaxia Reservoir on the upper Yellow River | 2023 | Chemosphere | Liujiaxia reservoir |

| | | | | |
|---|---|---|---|---|
| Bhola et.al. | Morphology and polymeric composition-based source apportionment of microplastics in surface water and sediment of drinking water supply reservoirs in Ranchi, India | 2024 | Environmental Processes | Getalsud reservoir; Dhurwa reservoir; Kanke reservoir |
| Di et.al. | Microplastics in surface waters and sediments of the Three Gorges Reservoir, China | 2018 | Science of the Total Environment | Three Gorges dam reservoir |
| Kumayon et.al. | Levels and composition of microplastics and microfibers in the South Saskatchewan River and stormwater retention ponds in the City of Saskatoon, Canada | 2023 | Environmental Science and Pollution Research | Gardiner dam |

| | | | | |
|---|---|---|---|---|
| Dhivert et.al. | Microplastic trapping in dam reservoirs driven by complex hydrosedimentary processes (Villerest Reservoir, Loire River, France) | 2022 | Water Research | Villerest reservoir |
| Vayghan et.al. | Spatial distribution of microplastics pollution in sediments and surface waters of the Aras River and reservoir: An international river in Northwestern Iran | 2022 | Science of the Total Environment | Aras reservoir |
| Di et.al. | Pollution in drinking water source areas: microplastics in the Danjiangkou Reservoir, China | 2019 | Environmental Toxicology and Pharmacology | Danjiangkou reservoir |
| Truong et.al. | Abundance of microplastics in surface water of tropical reservoirs during | 2024 | International Journal of Environmental Science and Technology | Dau Tieng reservoir; Tri An reservoir |

| | | | | |
|---|---|---|---|---|
| | contrasted season, the case of Dau Tieng and Tri An, Vietnam | | | |
| Huang et.al. | Coupled effects of urbanization level and dam on microplastics in surface waters in a coastal watershed of Southeast China | 2020 | Marine Pollution Bulletin | Shuikou reservoir |
| Queiroz et.al. | Implications of damming and morphological diversity of microplastics in the sediment from a tropical freshwater reservoir | 2024 | Journal of Environmental Chemical Engineering | Billings reservoir |
| Strojny et.al. | Preliminary study of the occurrence of microplastics in the sediments of the Rzeszów reservoir using the Laser Direct Infrared (LDIR) method | 2023 | Sustainability | Rzeszów reservoir |

| | | | | |
|---|---|---|---|---|
| Hübner et.al. | Microplastic concentrations at the water surface are reduced by decreasing flow velocities caused by a reservoir | 2020 | Fundamental and Applied Limnology | Rieselfelder reservoir |
| Baldwin et.al. | Microplastics in Lake Mead national recreation area, USA: occurrence and biological uptake | 2020 | PLOS One | Hoover dam |
| Turhan et.al. | Evaluation of microplastics in the surface water, sediment and fish of Sürgü dam reservoir (Malatya) in Turkey | 2021 | Turkish Journal of Fisheries & Aquatic Sciences | Sürgü reservoir |
| Nousheen et.al. | Comprehensive analysis of spatial distribution of microplastics in Rawal Lake, Pakistan using trawl net and sieve sampling methods | 2022 | Chemosphere | Rawal Lake |

| | | | | |
|---|---|---|---|---|
| Nocoń et.al. | Changes in the microplastic content depending on the changes in the river catchment development structure–preliminary studies | 2023 | Desalination and Water Treatment | Wisła-Czarne dam reservoir |
| Mo et.al. | Characteristics and ecological risks of microplastic pollution in a tropical drinking water source reservoir in Hainan province, China | 20241 | Environmental Science Processes & Impacts | Chitian reservoir |
| Shruti et.al. | Microplastics in freshwater sediments of Atoyac River basin, Puebla City, Mexico | 2019 | Science of the Total Environment | Valsequillo reservoir |
| Zhang et.al. | Vertical differentiation of microplastics influenced by thermal stratification in a deep reservoir | 2023 | Environmental Science & Technology | Tankeng reservoir |

| | | | | |
|---|---|---|---|---|
| Park et.al. | Temporal and spatial distribution of microplastic in the sediment of the Han River, South Korea | 2023 | Chemosphere | Paldang reservoir |
| Wu et.al. | Effect of cascade damming on microplastics transport in rivers: a large-scale investigation in Wujiang River, Southwest China | 2022 | Chemosphere | Wujiang reservoir |
| He et.al. | Effects of cascade dams on the occurrence and distribution of microplastics in surface sediments of Wujiang river basin, Southwestern China | 2022 | Ecotoxicology and Environmental Safety | Wujiang reservoir |
| Liu et.al. | Environmental fate of microplastics in alpine and canyon-type river-cascade | 2024 | Science of the Total Environment | Yalong River basin |

| | | | | |
|---|---|---|---|---|
| | reservoir systems: large-scale investigation of the Yalong River in the eastern Qinghai-Tibet Plateau | | | |
| Shen et.al. | Damming has changed the migration process of microplastics and increased the pollution risk in the reservoirs in the Shaying River Basin | 2023 | Journal of Hazardous Materials | Shaying river basin |
| Watkins et.al. | The effect of dams on river transport ofmicroplastic pollution | 2019 | Science of the Total Environment | Six Mile dam; Fall Creek dam |
| Ilmi et.al. | Microplastic risk assessment in river sediments along the cascading dam system (case study: midstream of the Citarum River, Indonesia) | 2023 | Water, Air, & Soil Pollution | Citarum River reservoir |

| | | | | |
|---|---|---|---|---|
| Pojar et.al. | Microplastic evaluation in water and sediments of a dam reservoir – riverine system in the eastern carpathians, Romania | 2024 | Sustainability | Buzău River–Siriu reservoir |

**Appendix S2. Bibliography of studies included in the meta-analysis of microplastics effect on freshwater benthic organisms.**

| **Authors** | **Title** | **Year** | **Journal** | **Species** |
|---|---|---|---|---|
| Lu et.al. | Metabolomic responses in freshwater benthic invertebrate, *Chironomus tepperi*, exposed to polyethylene microplastics: A two-generational investigation | 2023 | Journal of Hazardous Materials | *Chironomus tepperi* |
| Schell et.al. | Effects of polyester fibers and | 2022 | Environmental Toxicology and | *Daphnia magna; Asellus* |

| | | | | |
|---|---|---|---|---|
| | car tire particles on freshwater invertebrates | | Chemistry | *aquaticus; Hyalella Azteca; Lumbriculus variegatus* |
| Au et.al. | Reponses of *Hyalella azteca* acute and chronic microplastic exposures | 2015 | Environmental Toxicology and Chemistry | *Hyalella azteca* |
| Redondo-Hasselerharm et.al. | Ingestion and chronic effects of car tire tread particles on freshwater benthic macroinvertebrates | 2018 | Environmental Science & Technology | *Gammarus pulex; Asellus aquaticus; Tubifex spp.; Lumbriculus variegatus* |
| Lei et.al. | Microplastic particles cause intestinal damage and other adverse effects in zebrafish *Danio rerio* and nematode | 2018 | Science of the Total Environment | *Caenorhabditis elegans* |

| | | | | |
|---|---|---|---|---|
| | *Caenorhabditis elegans* | | | |
| Nugnes et.al. | Toxic impact of polystyrene microplastic particles in freshwater organisms | 2022 | Chemosphere | *Heterocypris incongruens* |
| Silva et.al. | Effects of polyurethane small-sized microplastics in the Chironomid, *Chironomus riparius*: responses at organismal and sub-organismal levels | 2022 | International Journal of Environmental Research and Public Health | *Chironomus riparius* |
| Redondo-Hasselerharm et.al. | Microplastic effect thresholds for freshwater benthic macroinvertebrates | 2018 | Environmental Science & Technology | *Gammarus pulex; Asellus aquaticus; Sphaerium corneum; Hyalella Azteca; Tubifex spp.;* |

| | | | | |
|---|---|---|---|---|
| | | | | *lumbriculus variegatus* |
| Ruijter et.al. | Microplastic effect tests should use a standard heterogeneous mixture: multifarious impacts among 16 benthic invertebrate species detected under ecologically relevant test conditions | 2023 | Environmental Science & Technology | *Gammarus pulex; Hyalella azteca; Asellus aquatics; Sphaerium corneum; Corbicula fluminalis; Potamopyrgus antipodarum; Tubifex spp.; Lumbriculus variegatus; Chironomus riparius* |
| Khosrovyan et.al. | Evaluation of the hazard of irregularly-shaped co-polyamide microplastics on the freshwater non-biting midge *Chironomus riparius* through its | 2020 | Chemosphere | *Chironomus riparius* |

| | | | | |
|---|---|---|---|---|
| | life cycle | | | |
| Kumari et.al. | Microplastics affect rates of locomotion and reproduction via dietary uptake in globally invasive snail *Physa acuta* | 2023 | Water | *Physa acuta* |
| Silva et.al. | Microplastics altered cellular responses, physiology, behavior, and regeneration of planarians feeding on contaminated prey | 2023 | Science of the Total Environment | *Chironomus riparius* |
| Silva et.al. | Ingestion of small-sized and irregularly shaped polyethylene microplastics affect *Chironomus riparius* life- | 2019 | Science of the Total Environment | *Chironomus riparius* |

| | | | | |
|---|---|---|---|---|
| | history traits | | | |
| Romero-Blanco et.al. | Assessment of the effects of environmental concentrations of microplastics on the aquatic snail *Potamopyrgus antipodarum* | 2021 | Water Air, & Soil Pollution | *Potamopyrgus antipodarum* |
| Ziajahromi et.al. | Environmentally relevant concentrations of polyethylene microplastics negatively impact the survival, growth and emergence of sediment-dwelling invertebrates | 2018 | Environmental Pollution | *Chironomus tepperi* |
| Edo Varg et.al. | Single and combined effects of | 2021 | Environmental Pollution | *Chironomus riparius* |

| | | | | |
|---|---|---|---|---|
| | microplastics, pyrethroid and food resources on the life-history traits and microbiome of *Chironomus riparius* | | | |
| Pedersen et.al. | Microplastic ingestion by quagga mussels, *Dreissena bugensis*, and its effects on physiological processes | 2020 | Environmental Pollution | *Dreissena bugensis* |
| Setyorini et.al. | Transfer and effects of PET microfibers in *Chironomus riparius* | 2021 | Science of the Total Environment | *Chironomus riparius* |
| Hallai et.al. | Understanding the microplastic pollution impact on *Chironomus* | 2024 | Knowledge & Management of Aquatic Ecosystems | *Chirinomus sancticaroli* |

| | | | | |
|---|---|---|---|---|
| | *sancticaroli* larvae development and emergence | | | |

## References


Borenstein, M., Hedges, L.V., Higgins, J.P., Rothstein, H.R., 2021. Introduction to meta-analysis. John Wiley & Sons.

De Graaff, M.A., Van Groenigen, K.J., Six, J., Hungate, B., Van Kessel, C., 2006. Interactions between plant growth and soil nutrient cycling under elevated $CO_2$: a meta-analysis. Glob. Change Biol. 12 (11), 2077-2091. https://doi.org/10.1111/j.1365-2486.2006.01240.x.

Egger, M., Smith, G.D., Schneider, M., Minder, C., 1997. Bias in meta-analysis detected by a simple, graphical test. bmj 315 (7109), 629-634. https://doi.org/10.1136/bmj.315.7109.629.

Gurevitch, J., Hedges, L.V., 2020. Meta-analysis: combining the results of independent experiments, Design and analysis of ecological experiments. In S. M. Scheiner & J. Gurevitch (Eds.), Design and analysis of ecological experiments (pp. 378–398). Chapman and Hall/CRC.

Hedges, L.V., 1981. Distribution theory for Glass's estimator of effect size and related estimators. J. Educ. Stat. 6 (2), 107-128. https://doi.org/10.3102/10769986006002107.

Koricheva, J., Gurevitch, J., Mengersen, K., 2013. Handbook of meta-analysis in ecology and evolution. Princeton University Press.

Moher, D., Liberati, A., Tetzlaff, J., Altman, D. G., & PRISMA Group., 2009. Preferred reporting items for systematic reviews and meta-analyses: the PRISMA statement. PLoS Med. 6 (7), e1000097. https://doi.org/10.1371/journal.pmed.1000097.

Pullin, A.S., Stewart, G.B., 2006. Guidelines for systematic review in conservation and environmental management. Conserv. Biol., 20 (6), 1647–1656. https://doi.org/10.1111/j.1523-1739.2006.00485.x.

Viechtbauer, W., 2007. Accounting for heterogeneity via random-effects models and moderator analyses in meta-analysis. Zeitschrift für Psychologie / J. Psychol., 215 (2), 104–121. https://doi.org/10.1027/0044-3409.215.2.104.

Viechtbauer, W., 2010. Conducting meta-analyses in R with the metafor package. J. Stat. Softw., 36 (3), 1-48. https://doi.org/10.18637/jss.v036.i03.

Violle, C., Navas, M.L., Vile, D., Kazakou, E., Fortunel, C., Hummel, I., Garnier, E., 2007. Let the concept of trait be functional! Oikos 116, 882-892. https://doi.org/10.1111/j.2007.0030-1299.15559.x.
